# Practical Guidance for Implementing Predictive Biomarkers into Early Phase Clinical Studies

**DOI:** 10.1155/2013/891391

**Published:** 2013-10-22

**Authors:** Matthew J. Marton, Russell Weiner

**Affiliations:** Merck Research Laboratories, Clinical Biomarkers and Diagnostics Laboratory, 126 E. Lincoln Avenue, Rahway, NJ 07065, USA

## Abstract

The recent U.S. Food and Drug Administration (FDA) coapprovals of several therapeutic compounds and their companion diagnostic devices (FDA News Release, 2011, 2013) to identify patients who would benefit from treatment have led to considerable interest in incorporating predictive biomarkers in clinical studies. Yet, the translation of predictive biomarkers poses unique technical, logistic, and regulatory challenges that need to be addressed by a multidisciplinary team including discovery scientists, clinicians, biomarker experts, regulatory personnel, and assay developers. These issues can be placed into four broad categories: sample collection, assay validation, sample analysis, and regulatory requirements. In this paper, we provide a primer for drug development teams who are eager to implement a predictive patient segmentation marker into an early clinical trial in a way that facilitates subsequent development of a companion diagnostic. Using examples of nucleic acid-based assays, we briefly review common issues encountered when translating a biomarker to the clinic but focus primarily on key practical issues that should be considered by clinical teams when planning to use a biomarker to balance arms of a study or to determine eligibility for a clinical study.

## 1. Introduction

At many biopharmaceutical companies, predictive biomarker assays are developed and validated either internally or externally with partner companies with expertise in molecular analyses. In either case, a multidisciplinary internal biomarker team will be needed to define assay requirements, select a diagnostic company partner, develop a workplan, and oversee the assay development and validation processes (reviewed in [1]). The team typically includes representatives from various departments, such as preclinical development, the clinical therapeutic area, Program management, Regulatory Affairs, clinical statistics, and Companion Diagnostics. Regular team meetings are highly recommended and are intended in part to facilitate communication and to ensure the team is able to adapt to both major changes (such as a change in clinical development timeline or target indication) and minor changes (such as changes to the list of clinical sites or to the specimen collection method) that may affect assay development or validation. 

## 2. Sample Collection Considerations

### 2.1. Sample Collection Method

After the identification of the biomarker and the source tissue from which the predictive biomarker will be assayed, the next most important consideration is how the sample will be collected and preserved in the clinical setting. Four key guiding principles are the following: (1) the collection method should utilize noninvasive or minimally invasive techniques (e.g., blood, plasma, and hair follicles) instead of more invasive techniques (e.g., tissue biopsies), if possible, (2) the amount of specimen requested should be minimized, yet be sufficient for analysis and possible retesting of the specimen, (3) the collection and preservation method should be demonstrated to be technically and logistically feasible in a clinical setting before planning its use in a clinical study, and (4) the collection method should preserve the sample quality quickly and not introduce a sample collection bias, especially when the analyte is labile or sensitive to subtle changes in temperature or handling conditions. The final procedure for collecting, processing, storing, and shipping the clinical samples is typically documented in a Procedures Manual (also called Operations Manual), which is a set of detailed instructions for clinical sites and the central laboratory and which typically is a collaborative effort between the assay developer and the clinical research associate. Since the Procedures Manual is usually needed 1–3 months before the first patient is enrolled, it is important to establish the details of the collection method early in the process, typically during clinical protocol development. For common sample collection procedures, a standard method may already exist in the company's method repository, complete with a supply list and site training materials. However, for unusual methods or novel specimen types, or if multiple analytes need to be measured from the same specimen, the team should allow several months for the development and validation of a novel collection method that is appropriate for the clinic. For example, a recent protocol in our laboratory required from the same sample the analysis of multiple biomarkers (RNA, DNA, and protein) including one predictive biomarker to identify eligible patients. Because RNA is more labile than the other analytes, it was important to stabilize the RNA-based pharmacodynamic biomarker first, even though it was less crucial than the DNA-based patient selection biomarker and exploratory protein-based biomarker. This required the development of a complicated collection method in which the sample was split, one half immediately placed into preservative while the other was further processed. This also highlights the need for clinical biomarker teams to prioritize the desired biomarkers if the amount of specimen available becomes limiting. For complicated or unusual procedures done at the clinical site, it may be necessary to train personnel at the clinical site in person or to create visual aids for training (such as videos, slide presentations, and operations cards) and to perform a pilot experiment to qualify a site to do the procedure.

### 2.2. Importance of Retaining Samples for Potential Bridging Studies

In some programs, late phase studies may be supported using a clinical trial assay (CTA; see Table 1 for definitions), even though the drug will require an *in vitro* diagnostic (IVD) device at product launch. This might occur when a clinical team wants to quickly transition from a promising phase II study that used a CTA to a pivotal phase III study before the companion diagnostic is in its final form. In that case, it may be necessary to “bridge” results from the CTA or other prototype assays to the final assay by reanalyzing the samples using the assay in its final IVD configuration to support the diagnostic device Pre-Market Approval (PMA) filing. As discussed below in the Regulatory Considerations section, companion diagnostic teams should avoid relying on a bridging strategy if at all possible. But when this is necessary, it will be crucial to plan, even for early phase studies, to obtain enough samples so that some can be stored and then reanalyzed using the version of the assay that will be submitted for health authority approval. There are many challenges with a bridging approach. First, if the samples are stored prior to sample analysis, auditable chain-of-custody documentation and rigorous sample stability studies are likely to be required. This can be a significant amount of work, and it likely means that merely storing samples for bridging studies in a good laboratory practice (GLP) environment will be insufficient. Second, sample banking and retention can also be challenging since the FDA requires that 90–95% of samples be available for retesting if using the data to support a PMA filing. Third, and most significantly, discordant results between the CTA and the final assay are inevitable and introduce risk into the device approval, since unexplained discordant results could cause regulatory authorities to question the technical performance of the diagnostic device. 

### 2.3. Special Considerations for Formalin-Fixed Slide-Based Assays

Use of formalin-fixed paraffin-embedded (FFPE) tissue in a predictive biomarker assay introduces additional challenges, one of which is ensuring the stability of the analyte after sectioning until the sample is analyzed. This is a significant concern for immunohistochemical (IHC) assays since protein stability can vary considerably depending on the analyte [2]. If the analyte is unstable, variability in the time in storage or shipment can confound analytical results. Ideally, clinical sites would submit an entire block for the analysis so that the central lab can complete the sectioning and then perform the sample analysis in a controlled timeframe. However, this may not be possible, either because the original block cannot be found or because the site will not agree to send the entire block. If this is the case, it is recommended that a cut slide stability study be performed to assess the stability of the analyte in sectioned slides. If degradation of signal is observed in a timeframe less than that which is necessary to perform the analysis, it may be necessary to use freshly sectioned slides.

Another critical parameter for a FFPE-based patient selection assay, but one issue that sometimes is overlooked or given insufficient attention, is the minimum percent of tumor required for the assay. This can be difficult to determine empirically, but for prospective enrollment assays it is important to specify if there is a chance the CTA will progress to a companion diagnostic. For example, the cobas BRAF test only requires 5% tumor [3], whereas other assays specify a minimum of 30% tumor [4] or specify that specimens with tumor content below 50% should be macrodissected [5]. How percent of tumor is calculated should also be carefully defined, whether it will be by percent nuclei or percent area. When selecting clinical sites, teams should consider a site's ability to have slides marked by a licensed pathologist and its ability to perform macrodissection, in case that is necessary. When requesting FFPE sections, the best practice is to collect two additional sections (immediately before and after the section(s) being tested) that can be used for H&E staining to assess the tumor content and pathology of the sample analyzed. This is especially true for assays that require multiple sections (e.g., some RNA- or DNA-based assays require three 10-micron sections per assay).

### 2.4. Postcollection Handling and Shipping of Clinical Samples

 The details of the postcollection handling and sample shipping conditions should not be overlooked. For assays that require specialized processing after collection, such as *ex vivo* cytokine induction or purification of peripheral blood mononuclear cells (PBMCs), two key variables that need to be optimized and controlled are the time between collection and processing and the shipping temperature [6]. The team will have to weigh several factors to decide whether these preanalytical steps are performed locally or centrally. A central lab may have more carefully controlled procedures, but if the time needed to ship to the central lab can directly impact the biomarker, it may be necessary to consider the use of local labs. The challenge is that local labs frequently do not have the required expertise or equipment, and this likely means extra effort for training, on-site monitoring, and establishing quality-control procedures at each clinical site. (See the Sample Analysis Considerations section below for additional concerns about using local laboratories.) For highly sensitive, single cell-based assays such as ELI-spot assays, it may be possible to use cryopreserved samples but there is no shortcut to doing controlled sample shipping studies to determine whether whole blood can be used or whether samples should be cryopreserved prior to shipping. Shipping conditions can also affect the shipment of FFPE slides and blocks. Although they are typically shipped at ambient temperatures, samples preserved in paraffin are at risk for melting, especially in the summer months, due to hot seasonal weather or from high temperatures encountered in transit. The Centers for Disease Control and Prevention recommend that blocks or slides be shipped with a frozen gel ice-pack. For frozen specimens, it is reasonable to consider a combination of dry ice and frozen gel ice-packs if the shipment is expected to take more than several days, as the ice-packs will remain frozen after the dry ice has sublimated. The actual temperature experienced can be tracked by radio frequency identification (RFID-) enabled temperature-tracking devices that are built into a shipping container. Alternatively, low technology solutions, such as temperature indicator labels that change color if the temperature exceeds a preset limit, are an inexpensive investment to monitor the integrity of the clinical sample-containing shipment. 

## 3. Assay Considerations

### 3.1. General

**Figure 1:**
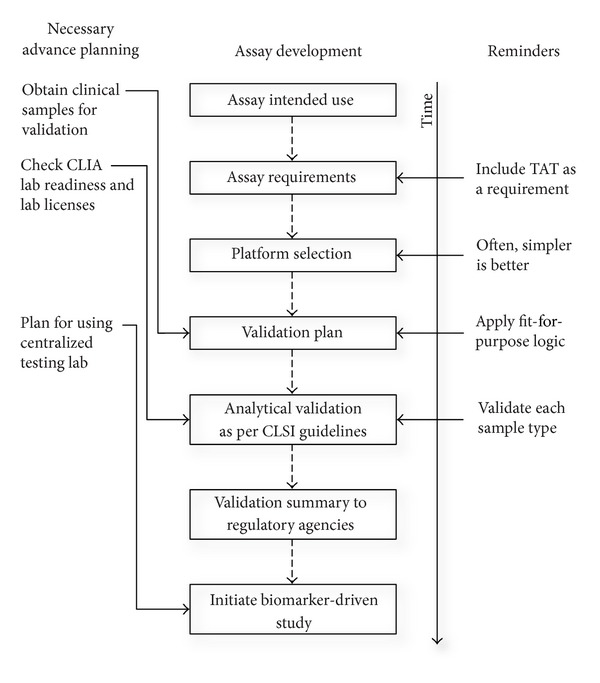
Schematic diagram of assay development activities. Development begins with defining the intended use, which is documented along with assay requirements. After platform selection, a validation plan is developed and executed according to CLSI guidelines. The validation summary is sent to the regulatory agencies prior to the initiation of the clinical study. Reminders discussed in the text are shown to the right. The relative timing of when steps that require advance planning should start is shown to the left.

Analytical validation always starts with intended use (Figure 1). It drives the development of the assay analytical validation plan. It is a good idea to document the intended use in some controlled document, such as an Assay Charter, which should describe the assay, the assay output, how “positive” and “negative” calls are made, and how the results will be used to determine patient eligibility. When a predictive marker will be used to direct patient enrollment or to balance arms of a study, the assay will need to be performed in a Clinical Laboratory Improvement Amendments (CLIA) laboratory. Since clinical labs are also regulated at the state level, it is possible that a “CLIA-certified” lab may not be certified to analyze samples from certain states. Therefore, it is important to confirm that the lab has the necessary certifications from each state where patients will be enrolled and to allow sufficient time for a newly certified CLIA lab to obtain all the needed state licenses. Most CLIA labs follow Clinical and Laboratory Standards Institute (CLSI) guidelines for determination of standard assay parameters such as precision, accuracy, limit of detection, specificity, and reference range. Although it is not unique to predictive biomarker assays, obtaining clinical specimens for assay validation and the determination of interpatient variability of the study population is critical to the success of the assay. When it is necessary to establish a threshold, that is, the clinical decision point, for a quantitative continuous biomarker, obtaining appropriate validation samples is frequently the rate limiting step in assay validation, so it is wise for the team to establish the strategy (purchase commercially or collaborate, e.g.) for obtaining the samples early in the assay validation phase. The rest of this section will highlight some concerns specific to predictive biomarkers.

### 3.2. Clinical Trial Assay Development Timeline

 It is important for the team to build adequate time into the schedule for assay development. The lead time for assay development and validation depends greatly on the assay platform and the complexity of the assay. This time could be as little as one month for an already-developed assay (e.g., a single prevalidated SNP TaqMan assay) to more than six months for a complex assay (e.g., multianalyte flow cytometry or microarray-based RNA expression signature) or for an assay that has not been deployed previously as a patient enrollment criterion. Even assays that may appear to be already validated can take a considerable amount of time to validate if the sample preservation method is changed. For example, when translating a microarray RNA expression signature from fresh frozen to FFPE specimens, different probes may need to be selected and validated, which essentially means that the assay has to be redeveloped before it can be validated [7]. Another factor that can impact assay timelines dramatically is the lead time for an agreement with a Testing Lab or diagnostic partner, which can easily add several additional months, especially if there are intellectual property issues to address. Finally, if the study plans to utilize a Testing Lab not previously used, the Testing Lab may need to undergo a more rigorous qualification or biosample handing audit for a predictive biomarker than may be necessary for other types of biomarkers.

### 3.3. Validation Strategy and Fit-for-Purpose Validation

 The assay development team should propose a plan of how to validate the clinical trial assay in the CLIA lab to the rest of the team for its input and feedback and to ensure alignment on the project specifics (sample type, collection method and any unique aspects of the biomarker). It is important to note that even if a predictive biomarker assay is developed and validated internally, the analytical validation of the assay will most likely have to be repeated in the CLIA lab supporting the clinical study. If the clinical trial assay will be assayed from more than one tissue, each sample type (e.g., tumor tissue, plasma, and bone marrow) will need to be validated since the preservation method may influence analyte abundance. Even though the predictive biomarker assay will have to be performed in a regulated laboratory, the fit-for-purpose concept is still applicable. Thus, the nature of the clinical study (e.g., phase I, II) and the extent to which the biomarker proof-of-concept has been established is taken into account when devising the validation plan. For a phase I study, one must plan the appropriate level of validation while avoiding overinvesting in an assay for a compound with an uncertain future (most compounds in phase I fail). For example, a common principle in analytical validation is to validate each specific tissue type and the specific population expected in the clinical study. However, some early phase oncology studies enroll patients with any tumor type, that is, what is sometimes called an all-comers study. In such studies, it is impractical to validate every possible tumor type. Thus, the analytical validation strategy must not only consider what is practical and the scientific value of a rigorous validation of each tumor type but also the cost, since sample acquisition and assay development costs to validate each tumor type can easily exceed one million US dollars. Such flexibility, however, does not apply to an *in vitro* diagnostic assay supporting a pivotal phase III study. In that case, every tumor type in the study must be analytically validated.

### 3.4. Assay Technology Selection and Assay Readout

There are significant tradeoffs between platforms when selecting the technology for a predictive biomarker assay, and some platforms are more technically difficult to validate than others. Take, for example, the choice of validating RNA expression levels by quantitative PCR (qPCR), microarrays, or next-generation sequencing. qPCR is more straightforward but becomes impractical when assaying many dozens of genes. One possible tradeoff is to reduce the number of genes in the RNA expression signature, even though this may introduce risk related to whether the smaller signature will be as predictive as the original, larger signature. Validation of a microarray or sequencing platform, on the other hand, is much more challenging but the breadth of data obtained offers the opportunity to refine the biomarker so that it is more predictive in a subsequent trial. The team will need to consider whether the benefit of one platform justifies the added complexity, especially if the platform is one that has not previously been used for predictive biomarkers. Even though several microarray-based RNA expression assays have been cleared by the FDA [8, 9], none have gone through the PMA process, and implementing them into a clinical protocol is still challenging currently. Thus, when there is a high confidence that the drug/assay combination will be successful and that a regulated device will be required, it may be wise to use the simplest technology. In this save vein, when developing one's own assay, it may be a good idea to avoid use of novel proprietary reagents or kits if there is concern about the supplier's ability to supply material consistently or to manufacture the material under GMP, which will be required if a companion diagnostic is required.

### 3.5. Using an FDA-Approved Diagnostic

 Just because an assay is an FDA-cleared or FDA-approved *in vitro* diagnostic device does not mean that it is validated for use as a clinical trial assay. Just like any other predictive biomarker assay, it must be validated for the specific intended use, that is, specific tissue type, specific patient population, and specific collection method. For example, a *KRAS* mutation test was approved for testing FFPE specimens from colorectal cancer patients [10]. Although FDA-approved, this test cannot be used as a predictive marker for blood specimens or for other tissue types without validation of the specific tissue or collection method. 

### 3.6. Next Generation Sequencing (NGS)-Based Predictive Biomarkers

 The clinical application of massively parallel sequencing, usually called next generation sequencing, presents many technical, operational, and regulatory challenges that are specific to the technology. In the context of early phase drug studies, the type of NGS assay most commonly deployed is one designed to direct patient treatment by detecting tumor sample mutations in dozens to hundreds of cancer-related genes. Although several guidance documents addressing these cancer gene panel assays have been published recently [11], noticeably absent is an FDA Guidance Document that defines analytical validation requirements to ensure accuracy of mutation calls. Furthermore, since each laboratory or company may have its own mutation calling algorithm, this lack of clarity means there is no consensus on how to ensure the reliability of mutation calls. Thus, it is first imperative to fully define and understand the mutation detection pipeline and quality control steps being used, especially if working with an external partner. Furthermore, consistent with one guidance document [11], both analytical validation studies and clinically actionable mutation calls should be confirmed with an orthogonal mutation calling technology. Gene panels may be the most used assay now, but it will be only a short time until data from whole exon sequencing or RNA-sequencing (RNA-Seq) tests will be used as predictive biomarkers. These assays generate much more data and thus raise issues surrounding patient consent and independent review board (IRB) approvals. For example, RNA-Seq assays are likely to require the same patient consent and IRB approval as genetic profiling since RNA-Seq enables determination of single nucleotide polymorphisms (SNPs), some of which are clinically actionable because they are strongly associated with disease susceptibility or progression. Likewise, it will be necessary to put into place unambiguous policies that clearly explain that how patient data will be handled and reported, especially for unintended findings from NGS studies.

## 4. Sample Analysis Considerations

The primary considerations in the sample analysis arena are turnaround time (TAT), cost and whether to use a local laboratory.

### 4.1. Turnaround Time

When considering TAT, it is important to focus on the total TAT from the patient's perspective, not just the TAT to perform the assay or the logistics involved to get the sample to the Testing Lab. For example, if an assay requires a previously prepared diagnostic sample (such as a formalin-fixed, paraffin-embedded block), it may take several weeks to obtain the block for sample analysis from the local hospital if it was collected there. A TAT greater than two weeks may have an adverse effect on patient recruitment due to fierce competition for patients eligible for clinical studies and because some diseases (such as various leukemias) can progress very rapidly. Therefore, it is critical to understand how delays can impact the ultimate stakeholders, that is, patients and physicians. The clinical team should think through the logistic details of the end-to-end process, from sample acquisition, pathological analysis at the clinical site (H&E and/or macrodissection, if applicable), shipment to central laboratory (if applicable), shipment to CLIA laboratory, and workflow at the CLIA lab through the sending of the patient test report to the clinical site. It can be informative to ask the Testing Lab to provide a detailed hour-by-hour workflow of sample analysis as a way to spark discussions of how to reduce TAT. For mutation detection or gene expression assays, depending on the assay, the Testing Lab may need up to 10 business days to perform the assay and report results. Assays that require macrodissection of FFPE slides may take even longer time. To expedite TAT, the team can consider (1) sending the specimen directly to the Testing Lab instead of first sending to a central lab for sample accounting, (2) asking the Testing Lab to accept Saturday shipments or work on weekends, or (3) asking the Testing Lab to arrange shifts to accommodate a longer workday. Although in some cases it may be worth establishing multiple Testing Labs in different geographic regions to reduce TAT, in general, this may not impact TAT unless overnight shipment from the clinical site to the Testing Lab is unavailable.

### 4.2. Use of Local Laboratories

When is it acceptable to use an assay performed at a local laboratory for patient eligibility decisions? In general, teams should use assays validated in a centralized Testing Lab instead of assays performed at local labs (e.g., hospital labs) for eligibility decisions. Performing the assay at the local lab may have the benefit of shorter TAT but can have the liability of having greater variability resulting from (1) different laboratory methods or instruments, (2) different validation standards and quality control processes, (3) different histopathological practices in the macrodissection of tumor from nontumor, and (4) lab-to-lab variability due to a subjective or difficult-to-standardize assay (e.g., IHC). These concerns even apply to common assays (such as *KRAS* and *EGFR* mutation detection and Ki-67 IHC) and may result in discordant results. For example, a 2006 study showed a high degree of discordance between HER2 IHC results from a centralized lab compared to data from local labs at clinical sites [12]. More recently, André et al. (2013) reported results of retrospective analyses of a phase II study in which local labs were used to detect *KRAS* mutations to determine colorectal cancer patient eligibility for treatment with an anti-EGFR antibody. The authors found that 6 of the 60 enrolled patients had *KRAS* mutations and should have been excluded from the study [13]. Furthermore, an international study to assess proficiency of 59 European Testing Labs for *KRAS* mutation detection found that 31% of labs made miscalls in at least 10% of the samples [14]. Another reason to avoid using a local laboratory is that it is very likely that the FDA will question the merging of data generated with two different assays to support a filing. Despite these caveats, there may be cases when the use of a local lab for clinical trial assay is acceptable: (1) when the team is sure it does not want to use the data to support drug efficacy, and (2) when the test is an approved *in vitro* diagnostic, for example, when a clinical study's eligibility criteria requires that a patient's tumor harbor certain mutations and there already exists an approved or cleared *in vitro* diagnostic. In general, the risks of using a local lab will usually outweigh the benefits. Thus, it is recommended that molecular pathological analysis be performed at a central lab rather than at the individual clinical sites.

### 4.3. Cost

 The sample analysis cost for a complex predictive marker such as a RNA expression signature or a mutation detection panel in a clinical study can exceed $2000 per sample. Therefore, it is usually imperative that options be explored to reduce costs. This can be particularly challenging, especially for studies in which a clinical team expects that only a few patients will be enrolled per week or per month, which may mean that many samples will be analyzed individually. Therefore, teams should consider (and discuss with the FDA) alternate strategies for assay process controls to reduce the ratio of number of controls to number of samples. Also, if the Testing Lab assay time is 3 days or less, one should consider having the Testing Lab batch samples (e.g., only running the assay twice a week) if that would reduce cost while still providing acceptable TAT. Finally, if enrollment eligibility is dependent on two distinct predictive biomarkers assays, such as a qPCR assay and an IHC assay, one should consider whether there is an opportunity to perform the assays sequentially so that the second assay is performed only if the first indicates the patient is eligible.

### 4.4. Impact of Screen Failures on Enrollment

The clinical team should estimate and document the expected screen failure rate, and when projecting number of patients it will be necessary to screen, keeping in mind the difference between the percentage of patients deemed ineligible due to the test and the overall clinical study screen failure rate. The Testing Lab may need to know this number to adequately project the number of assays that will need to be performed and the amount of reagents that will need to be qualified. Doing this exercise early may help determine whether the enrollment strategy is appropriate and whether the clinical decision threshold (eligible/not eligible) is set appropriately.

## 5. Regulatory Considerations

### 5.1. Predictive Biomarker Tests Are under the Oversight of Centers for Medicare & Medicaid Services (CMS) and State Laws

U.S. Federal laws (CLIA '88) established quality standards for any laboratory that performs testing on human specimens for the purpose of diagnosing, or treating, or assessing patient health. Thus, predictive biomarker tests that are used to balance arms of a study or to select patients for enrollment into a clinical study are under the purview of both the CMS and the U.S. FDA. Labs must be CLIA-certified by the state in which they reside or by a CMS-approved accrediting institution such as the College of American Pathologists (CAP). In addition, some states have additional laws regulating in-state clinical laboratories or the analysis of their residents' samples, independent of where the analysis is conducted. Two states, New York and Washington, developed their own set of regulations for clinical labs, and CMS have deemed their states' clinical laboratories to have met CLIA requirements (i.e., they have been granted “deemed status”) because CMS has judged their state-specific regulations as equal to or exceeding CLIA standards. These and other states (CA, FL, MD, RI, and PA) have various regulations that may require one or more of (1) in-person inspection, (2) approval of validation report, (3) approval of laboratory SOPs, (4) proof of adequate lab personnel training and (5) lab management background checks. These regulations are put in place to protect patients treated in those states, so it matters more where the patients are treated than where the test is performed. New York, California, and Florida are known to have the strictest regulations, so if a planned clinical site is located in one of these states, the team must ensure the Testing Lab has those state certifications. It must be noted that in some cases there is some ambiguity on whether these state-specific regulations apply to clinical studies; the conservative approach is to ensure the lab has the state certification. The practical advice on this point is to engage the laboratory early and to realize that licensure may take up to 6 months. Also, although CLIA applies to laboratories located within the United States, Testing Labs outside the U.S. can request CLIA certification from various accrediting bodies if they plan to test samples from US citizens.

### 5.2. Predictive Biomarker Tests Are Laboratory-Developed Tests (LDTs) and Are under the Oversight of the FDA

Historically, LDTs were primarily niche assays, that is, highly specialized, low volume tests that were developed and validated in one clinical laboratory and which were not carefully monitored by the FDA. In FDA language, the FDA exercised enforcement discretion. Today, however, many clinical laboratories have developed predictive biomarker tests that are being used to direct patient treatment. A 2011 guidance document [15] and recent public statements from the FDA commissioner indicate that the agency regards these tests as *in vitro* diagnostics that need to go through the 510 (k) premarket notification or premarket approval (PMA) process. Furthermore, the guidance said the FDA will focus initially on high complexity testing assays, such as Multivariate Index Assays [16], assays which measure multiple analytes and use mathematical algorithms to determine the clinical significance of the test result. Thus, the regulatory compliance of predictive biomarkers involving multigene signatures is likely to be the focus of the most scrutiny by the FDA in the near term. This underscores the wisdom of discussing the assay and its analytical validation with the FDA prior to its use as a patient enrollment assay.

### 5.3. Laboratory-Developed Tests (LDTs) and *In Vitro* Diagnostics Devices (IVDs)

 A comprehensive discussion of LDTs and IVDs is beyond the scope of this paper, so only a few comments will be made here. The FDA recognizes two categories of IVD devices: a “kit” that is shipped from an IVD manufacturer to any appropriate clinical laboratory for use as a diagnostic or a laboratory-developed test (LDT), which can be thought of as a “service” offered by a single specific laboratory, within which the assay was developed, manufactured, and validated using specific equipment. In fact, the FDA considers any test used to direct patient treatment (including selecting patients for enrollment or balancing arms of a study) to be an IVD device that must be reviewed by the FDA [17]. Thus, both types of IVD devices must be reviewed and become either FDA-cleared (if submitted as a 510 (k) application) or FDA-approved (if submitted as a PMA application). Examples of FDA-cleared LDTs include the Tissue of Origin (TOO) test developed by Pathwork Diagnostics and the Mammaprint test developed by Agendia. A presubmission meeting should be planned with the FDA prior to introducing a predictive biomarker assay into a clinical study [17]. The submitted document should include items such as intended use, assay description, and rationale for use and should summarize preclinical studies supporting the assay's use. In addition, it should contain a risk analysis and analytical validation data documenting assay performance characteristics such as accuracy, precision, and linearity.

In summary, it is an exciting time for those involved in predictive biomarker research. Biomarker hypotheses are actively being prospectively tested in clinical studies. Despite the challenges outlined in this paper, stratified medicine is becoming a reality. It is our hope that our suggestions, recommendations and checklist (Table 2) contribute in some small way to the broader effort of fellow translational scientists in the development of stratified treatments that will tangibly benefit our patients.

## Figures and Tables

**Table 1:** Definitions.

Clinical Trial Assay (CTA): a predictive biomarker assay that is either: (1) a prototype form of a planned IVD kit, or (2) a laboratory-developed test that will not be commercialized and sold as a kit to other labs. If the CTA is essential for safe and effective use of the drug, then it must be bridged to a companion diagnostic.	
Laboratory-Developed Test (LDT): an *in vitro *diagnostic test that is developed, validated and used exclusively for in-house diagnostic purposes.	
*In vitro* diagnostic (IVD): any “device” intended for use in the diagnosis of disease or other condition, or in the cure, mitigation, treatment, or prevention of disease, in man or other animals. These devices must be cleared by the FDA through either the 510 (k) premarket notification process or must be approved through the PreMarket Approval (PMA) processes [17].	
Companion Diagnostic: an *in vitro* diagnostic device that provides information that is essential for the safe and effective use of a corresponding therapeutic product [17] and that will be commercialized along with the therapeutic. In general, this test must be clinically validated along with the drug in the registrational trials.	
Investigational Use Only (IUO): a regulatory term for a medical device undergoing validation in a clinical trial. A companion diagnostic is labeled as IUO while used in a registrational clinical trial [15].	

**Table 2:** Predictive biomarker checklist.

	*Team formation *
□	Form team; include representatives from assay development, clinical therapeutic area, program management, regulatory affairs and clinical statistics
□	Establish regular team meetings
	*Sample collection considerations *
□	Determine source tissue for biomarker analysis
□	Minimize amount of specimen required; use non-invasive techniques if possible
□	Allow 1–3 months if sample collection method does not exist
□	Train personnel at clinical site, if needed; create visual aids for training
□	Retain extra specimens for potential bridging studies
□	If utilizing a bridging strategy, initiate sample stability studies
□	If using FFPE specimens, establish minimum percent tumor specification
□	Select clinical sites with licensed pathologist able to mark slides and perform macrodissection
□	Collect extra sections before and after sections being analyzed for H&E staining
□	Perform sample collection experiment to qualify each clinical site, if necessary
	*Assay considerations *
□	Clearly define and document assay intended use, how positive and negative calls are made and how results determine patient eligibility
□	Select assay technology platform, consider assay output, establish clear requirements
□	Develop validation strategy, validating each sample type or collection method
□	Allow several months to complete vendor agreement
□	Allow time for vendor qualification, if new vendor
□	Obtain clinical specimens for analytical validation and decision-point threshold
□	Allow 1–6 months for assay development for a CTA; at least 24 months for a IVD
	*Sample analysis considerations *
□	Document anticipated turn around time from patients' perspective
□	Document sample logistics from acquisition to patient test report
□	Request hour-by-hour workflow of assay from vendor
□	Have clinical site send specimen directly to testing lab if possible
□	Ask vendor to accept Saturday shipments, to work weekends or to work longer days
□	Avoid the use of local labs
□	Reduce cost by batching samples, for example, biweekly sample analysis
□	Explore alternate control strategies to reduce cost of running process controls
□	Consider performing assays sequentially if using multiple predictive markers
□	Calculate and document anticipated screen failure rate
	*Regulatory considerations *
□	Identify CLIA lab with appropriate state licenses or allow 1–6 months for lab to obtain necessary licenses
□	Discuss high complexity assays with FDA before implementing in clinical trials
□	Set up pre-submission meeting with FDA in advance of clinical trial
□	For companion diagnostic development, work closely with partner on timelines
